# Multiplex on-chip detection of *Aspergillus* by integrated ultrasonication-based bead beating lysis and magnetic beads direct amplification

**DOI:** 10.3389/fbioe.2026.1775828

**Published:** 2026-02-19

**Authors:** Jinyu Zhong, Shiliang Zhang, Wei Huang, Jie Cheng, Xiaoning Li, Sen Wang, Tong Zhang, Guodong Sui

**Affiliations:** 1 Shanghai Key Laboratory of Atmospheric Particle Pollution and Prevention (LAP3), Department of Environmental Science and Engineering, Fudan University, Shanghai, China; 2 Shanghai Sci-Tech Inno Center for Infection and Immunity, Shanghai, China; 3 Department of Clinical Laboratory, Shanghai East Hospital, Shanghai, China; 4 Department of Clinical Laboratory, The Fifth People’s Hospital of Wuxi, Wuxi, China; 5 IngeDx Technologies Co., Ltd., Suzhou, China; 6 Department of Infectious Diseases, Shanghai Key Laboratory of Infectious Diseases and Biosafety Emergency Response, National Medical Center for Infectious Diseases, Huashan Hospital, Shanghai Medical College, Fudan University, Shanghai, China; 7 Shanghai Institute of Infectious Disease and Biosecurity, Fudan University, Shanghai, China

**Keywords:** *Aspergillus fumigatus*, beads beating, magnetic beads direct amplification, thin-film microfluidic chip, ultrasonication lysis

## Abstract

**Introduction:** Invasive aspergillosis (IA) is a life-threatening disease in immunocompromised individuals, creating an urgent need for rapid, sensitive, and user-friendly methods for early detection of *Aspergillus* spores in clinical and environmental samples.

**Methods:** In this study, we developed a rapid ultrasonication-based beads beating (USBB) method for cell lysis and fungal DNA release, along with an integrated magnetic beads-based direct amplification (MBDA) method utilizing microfluidic chip technology. Our flexible thin-film microfluidic chip was developed to enable immediate contacting with an ultrasonic oscillator for nucleic acid extraction.

**Results and discussion:** The chip achieved a lysis efficiency of 85.5% for *Aspergillus* at 10^5^ spores per sample. Magnetic beads releasing Fe ions at 75-860 ng/mL allowed high-efficiency direct PCR amplification. Complete transfer of the nucleic acid extract into PCR amplification led to a 100-fold improvement in detection sensitivity. The integrated USBB-MBDA system completed the entire workflow from sample-in to result-out within 35 min (5 min fungal DNA release and 30 min TaqMan assay). Using this approach, we achieved simultaneous triplex detection of *Aspergillus* *fumigatus, Aspergillus flavus*, and* Aspergillus niger*, with a sensitivity as low as 10 spores per test. Validation on 37 clinical samples showed complete concordance with MALDI-TOF mass spectrometry (MS). This study establishes an integrated nucleic acid extraction and magnetic bead–enabled direct amplification strategy, providing a versatile approach for the development of automated analytical platforms with broad applicability to *in vitro* diagnostics.

## Graphical Abstract

**Figure:**
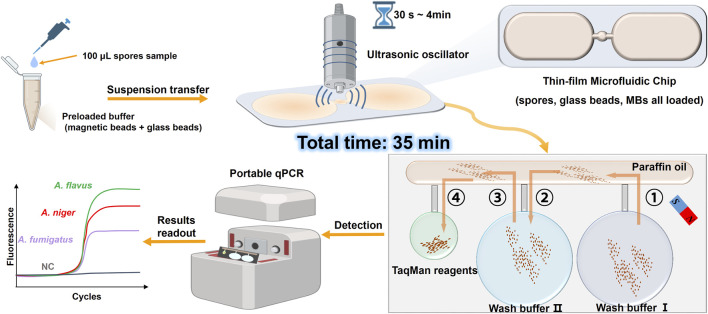
Detection of three invasive *Aspergillus* species on a microfluidic chip using ultrasonic beads beating for cell lysis combined with magnetic beads–based direct amplification.

## 1 Introduction

The global burden of invasive fungal infections (IFIs) reaches approximately 6.5 million cases annually, resulting in 3.8 million deaths (Denning, 2024). Invasive aspergillosis accounts for 2.11 million of these cases yearly, with a fatality rate of 85.2% (1.8 million deaths) (Denning, 2024; Hammond et al., 2020). However, IA incidence was estimated at only ∼200,000 annual cases in 2012, demonstrating a sustained upward trajectory globally (Garcia-Solache and Casadevall, 2010; Herrera et al., 2025). For immunocompromised individuals, exposure to *Aspergillus* spore-rich air significantly increases the risk of developing invasive aspergillosis through pulmonary inhalation, the primary infection pathway (Robin et al., 2019), causing asthma, allergic rhinitis, cutaneous hypersensitivity reactions and pulmonary aspergillosis (Bush et al., 2006). The most common causative agent of pulmonary aspergillosis is *Aspergillus fumigatus (A. fumigatus)*, followed by *Aspergillus flavus (A. flavus)* and *Aspergillus niger (A. niger*) (Kosmidis and Denning, 2015; Nucci and Anaissie, 2009). Besides causing respiratory symptoms, *A. fumigatus* can also lead to central nervous system (CNS) infections, resulting in headaches, altered consciousness, and other neurological manifestations (Muñoz et al., 2014; Nucci and Anaissie, 2009). *A. flavus* infection is particularly associated with acute hepatotoxicity and nephrotoxicity, necessitating avoidance of hepatotoxic agents during treatment (Falcone et al., 2011). *A. niger* may cause otomycosis (ear infections), often requiring local debridement (Chappe et al., 2018). Species identification of these three *Aspergillus* species enables targeted management and medication for IA patients. Thus, developing a rapid, sensitive, and user-friendly triplex detection method for *A. fumigatus*, *A. flavus*, and *A. niger* is of great practical value for early *Aspergillus* infection screening.

Currently, microscopic examination, tissue/secretion culture, serum antibody detection, and fungal antigen testing represent commonly utilized clinical diagnostic modalities for mycoses (Cadena et al., 2021). While microscopic is constrained by limited sensitivity and operator-dependent variability (Denning, 1998). Culture-based methods, regarded as the diagnostic gold standard, are hampered by prolonged incubation periods, suboptimal sensitivity, and frequent false-negative outcomes (Kullberg and Arendrup, 2015). The diagnostic accuracy of antibody and antigen detection assays is often compromised by false-negative or false-positive results, owing to individual heterogeneity in immune responses and serological cross-reactivity (Jenks et al., 2021; Lo Cascio et al., 2024; Schub et al., 2024). Nucleic acid amplification techniques (NAATs), such as Polymerase Chain Reaction (PCR), Rolling Circle Amplification (RCA), Recombinase Polymerase Amplification (RPA), Loop-Mediated Isothermal Amplification (LAMP), are dominating methods employed in research and clinical settings (Kang et al., 2022). Isothermal amplification techniques, like RCA, RPA and LAMP, are widely adopted for point-of-care testing (POCT) due to their minimal instrumentation requirements (Bi et al., 2025). However, these methods typically exhibit a limit of detection (LOD) of approximately 10 copies/reaction (Gu et al., 2025; Li et al., 2025; Wilkinson et al., 2024). In contrast, PCR achieves single-copy sensitivity, making it the preferred method for detecting ultra-low-abundance targets and the current gold standard in commercial diagnostic kits (Wang et al., 2019; Wang et al., 2026; Wang et al., 2022; Wee et al., 2020). During the COVID-19 pandemic, PCR technology achieved technological maturity characterized by its capacity to detect target sequences at ultra-low nucleic acid concentrations (approaching single-molecule levels) (Dong et al., 2021; Muhsin et al., 2023). And the utilization of species-specific primer-probe sets significantly enhances the application potential of PCR in detecting clinical samples with extremely low concentrations of *Aspergillus* infections.

The efficient extraction of pathogen genomes is a critical determinant of the overall analytical efficiency and diagnostic sensitivity. Compared with bacterial or viral pathogens, fungi possess a chitin-based rigid and thick cell wall structure, which significantly complicates genomic DNA extraction. Current strategies for fungal cell wall disruption primarily encompass enzymatic digestion (Borman et al., 2008; Jin et al., 2004; Springer et al., 2013), liquid nitrogen grinding (Bever et al., 2000), chemical lysis protocols (Lu et al., 2022; Vingataramin and Frost, 2015), and physical fragmentation methods (including thermal treatment (Baek et al., 2010; Koffi et al., 2023), electroporation (Kong et al., 2024), ultrasonication (Romanelli et al., 2014; Starke et al., 2019), and beads beating (Famà et al., 2025; Scharf et al., 2020). However, due to the structural heterogeneity of fungal cell walls, these methods cannot be widely applicable to all fungal groups. Almost all groups contain (1,3)-β-D-glucan and chitin as core components of their inner skeletal cell walls, as for *Aspergillus*, their hyphae possess an outer cell wall decorated with galactofuran-modified mannan chains, a hallmark of many filamentous fungi, forming a dense protective barrier (Erwig and Gow, 2016). Additionally, conidia of *Aspergillus* spp. are further characterized by a hydrophobic outer shell composed of α-1, 3-glucan and an inner melanin layer (Erwig and Gow, 2016), reinforcing their structural resilience. This distinctive morphological feature significantly impedes the efficacy of standard commercial DNA extraction kits.

In this study, we established a rapid fungal detection platform designated as the USBB-MBDA platform, which facilitates triplex detection of, *A. fumigatus*, *A. flavus*, and *A. niger*. The platform comprised two integrated modules: (1) an ultrasonication-based bead beating cell lysis module and (2) a magnetic beads (MBs)-based genomic DNA purification and direct amplification detection module (integrated on a microfluidic chip). This tube-based method achieved a LOD of 10 spores per test for three *Aspergillus* species in an end-to-end workflow completed within 110 min. While maintaining consistent sensitivity, we further integrated washing and amplification processes into a microfluidic chip, achieved the complete transfer of nucleic acids, eliminated complex pipetting and magnetic bead separation steps, avoided the contamination risk caused by frequent lid opening, and simultaneously realized a 100-fold enhancement in sensitivity compared with commercial kits. Integration onto a microfluidic platform enabled end-to-end processing (sample-in to result-out) within 35 min, demonstrating rapid analytical turnover performance which was critical for point-of-care diagnostics. Analytical performance of the USBB-MBDA system was further validated using 37 clinical samples, showing complete concordance with MALDI-TOF and achieving a sensitivity of 100% (95% CI: 87.2–100.0) and a specificity of 100% (95% CI: 69.2–100.0). To validate the system’s efficiency in detecting fungi that were challenging to extract, we successfully detected *Cryptococcus neoformans* (*C. neoformans*) with a LOD of 100 copies/test. In essence, this study developed an integrated nucleic acid extraction and magnetic bead-based direct amplification strategy, offering a versatile tool for developing automated analytical platforms with broad utility in *in vitro* diagnostics.

## 2 Materials and methods

### 2.1 Materials and reagents

The fungal strains *A. fumigatus* (CICC No.41022), *A. flavus* (CICC No.2436), *A. niger* (CICC 40048) and *C. neoformans* (ATCC 204092) were obtained from the China Center of Industrial Culture Collection (CICC). The actual clinical samples were kindly provided by Wuxi Fifth People’s Hospital and identified by MALDI-TOF mass spectrometry. DNeasy Blood & Tissue kit and MB 5 was purchased from QIAGEN (Hilden, Germany). TE buffer (pH 8.0), Magnetic Universal Genomic DNA Kit and MB 6 was purchased from Tiangen Biochemical Technology Co., Ltd. (Beijing China). MB 1, lysis buffer, binding buffer and wash buffer I/II was purchased from Novaz Bio-Technology Co., Ltd. (Nanjing China). MB 2-MB 4 was purchased from Enriching Biotechnology Co., Ltd. (Suzhou China). MB 7 was purchased from Beyotime Biotechnology Co., Ltd. (Shanghai China). Taq DNA Polymerase, 5 × Taq Buffer with Mg^2+^, and dNTPs were purchased from Novoprotein (Suzhou, China). Polymethyl Methacrylate (PMMA) boards were purchased from Jian chuan Technology Co., Ltd. (Wenzhou, China). Platemax UltraClear Sealing Film was purchased from Axygen (Union, United States). Zirconia beads, methyl red, paraffin liquid and other reagents were purchased from Aladdin (Shanghai, China).

### 2.2 Pathogenic fungi culture, collection and quantification

*A. flavus*, *A. niger*, and *A. fumigatus* were cultured on Salt Czapek-Dox Agar at 37 °C for 7–10 days to generate abundant conidia. The conidia were harvested by washing the cultures with 0.5% Tween-20 and resuspending in the same solution. *C. neoformans* was cultivated on Sabouraud’s Dextrose Agar at 37 °C for 5–7 days, and conidia were collected using phosphate-buffered saline (PBS). Fungal spore suspensions were quantified via hemocytometer and adjusted to 10^7^ spores/mL using 0.5% Tween-20 or PBS. All suspensions were stored at 4 °C and utilized within 2 weeks.

### 2.3 Development and validation of triplex TaqMan assays

Fungal standard genomes were extracted following the manufacturer’s protocol for the DNeasy Blood & Tissue Kit. TaqMan primer and probe sequences with species-specific nucleotide targets (*C. neoformans*: GenBank accession no. XM_770374, *A. fumigatus*: GenBank accession no. CP097568, *A. niger*: GenBank accession no. KX897144 and *A. flavus*: GenBank accession no. XM_041283833.1)) were designed using Beacon Designer 8 software (Premier Biosoft International, Palo Alto, CA, United States). The primers and probes were synthesized by General Biol (Anhui, China). Sequence details are summarized in Supplementary Table S1. To determine the specificity of the four primer-probe sets, cross-reactivity testing was performed against genomic DNA from *A. fumigatus*, *A. flavus*, *A. niger*, *C. neoformans*, and eight common respiratory pathogens respectively, using sterile water as a negative control. Three parallel experiments were carried out. Genomic DNA concentrations of the extracted fungi were quantified by NanoDrop™ One Microvolume UV-Vis spectrophotometer (Thermo Fisher Scientific, Waltham, MA, United States) (*A. fumigatus*: 7.3 ng/μL, *A. flavus*: 5.6 ng/μL, *A. niger*: 6.5 ng/μL, *C. neoformans*: 3.8 ng/μL). The DNA templates were serially diluted to achieve final concentrations ranging from 10^5^ to 1 copies/μL. Sensitivity analysis was performed using sterile water as negative control, with results visualized in Figure 2. TaqMan real-time PCR assay was performed in a 50 μL reaction solution containing 10 μL of sample, 5 × Taq Buffer with Mg^2+^(10 μL), 0.2 mM dNTPs(1 μL), 1.25 U Taq DNA Polymerase (0.25 μL), 0.4 μM forward primer (2 μL), 0.4 μM reverse primer (2 μL), 0.2 μM probe (1 μL), and 23.75 μL nuclease-free water. The mixture was incubated at 94 °C for 1 min, followed by 45 cycles of 20 s at 95 °C, 60 s at 60 °C. Taqman assay was conducted on an Applied Biosystems™ 7500 Real-Time PCR System (Thermo Fisher Scientific, Waltham, MA, United States).

### 2.4 The elution-free MBDA methods

To maximize nucleic acid utilization and avoid elution step, MBs, pre-bound with nucleic acids, were directly introduced into a 100 μL TaqMan mix system (The concentrations of the components were consistent with those in Section 2.3), ensuring complete using of extracted genomic DNA for amplification. The PCR procedure was the same as described above.

### 2.5 Establishment and evaluation of USBB fungal lysis methods

The customized ultrasonic system comprises three core components: an ultrasonic lysis module, an air control module, and a central control module (Figures 4A–C), with overall operation managed by a personal computer. The ultrasonic lysis module consists of dual gasbags controlled by the air control module and a circular metal ultrasonic oscillator between them (Figure 4D). During operation, the thin-film microfluidic chip (Figure 4E) is positioned over the lysis module to establish direct contact with both gasbags and the central ultrasonic oscillator. Under the regulation of the air control module, the gasbags undergo periodic inflation and deflation cycles, and the chip-contained biological fluid undergoes controlled mechanical compression, executing reciprocating motion (Supplementary Video S1). The oscillator emits a constant 20 kHz ultrasonic wave, which generates focused acoustic energy at its center. As the processed fluid passes through this oscillatory field, the microbial cell walls are subjected to cavitation-induced shear forces (Zhang et al., 2007), achieving cellular lysis. To further confirm the operational reliability of the ultrasonic system, its liquid mixing effect was validated using methyl red solution and TE buffer (Supplementary Video S1).

The DNA release efficiency of USBB for *Aspergillus* and *Cryptococcus* was evaluated at a concentration of 10^5^–10 spores/test. 100 μL of spore-containing sample was mixed with 500 μL lysis buffer, 200 μL binding buffer, 300 μL isopropanol, 200 μg MBs, and 0.05 g glass beads. The suspension was transferred to the thin-film microfluidic chip and sonicated for 1 min using the ultrasonic system. Subsequently, the mixture was transferred to a 1.5 mL EP tube. MBs were washed, air-dried, and resuspended in 100 μL pre-mixed TaqMan reaction reagents for further amplification on the Applied Biosystems™ 7500 Real-Time PCR System. The PCR procedure was the same as described as above. Two key variables—beads type and ultrasonication time—were further investigated to optimize the performance of the USBB system. For comparative analysis, two commercial DNA extraction kits were used as controls. Fungal DNA was extracted strictly following the manufacturers’ protocols and amplified using the same TaqMan assay program.

### 2.6 Competitive sensitivity assay of USBB-MBDA system

To determine the LOD of the USBB-MBDA system for one *Aspergillus* species in the presence of high concentrations of the other two species, three sets of verification experiments were designed. In each set, two species were spiked into the system at a high concentration of 10^4^ spores to act as competitive background, while the third target species was serially diluted from 10^4^ to 10^0^ spores and introduced into the system. Subsequent cell lysis and detection procedures were performed according to the method described in Section 2.5.

### 2.7 USBB-MBDA system evaluation in actual samples

The same procedure as described in 2.5 was applied to detect 37 actual samples collected from Wuxi Fifth People’s Hospital. All samples had been previously identified by MALDI-TOF MS. The results obtained by the USBB-MBDA were compared with the MALDI-TOF identification results to evaluate the feasibility of the USBB-MBDA system for clinical sample detection.

### 2.8 Microfluidic chip fabrication and operation

To integrate MBs washing procedures with the final amplification step, we designed and fabricated a three-layer microfluidic chip that consolidates these processes into an integrated platform. The 80 mm × 40 mm × 1 mm chip comprises: (1) a top PCR plate sealing film layer, (2) a 1 mm-thick polymethyl methacrylate (PMMA) chip housing four functional chambers (two 24-mm-diameter washing chambers, a 75 mm × 4 mm oil-phase chamber, and a 12-mm-diameter amplification chamber) interconnected by 1 mm-diameter channels (Figure 6A), and (3) a bottom PCR plate sealing film layer (Figure 6B). The PMMA chip was designed using AutoCAD™ (Autodesk, San Rafael, CA, United States) and fabricated via commercial microfabrication services. A 1 mm-diameter inlet on the oil-phase chamber’s sealing film serves as the injection port for liquid paraffin to establish a fully sealed system. During operation, pre-mixed TaqMan reagents, wash buffer II, and wash buffer I containing MBs are sequentially injected into the chambers, followed by liquid paraffin to seal the channel. Guided by external magnetic fields, the MBs follow a predefined trajectory (washing chamber I → oil-phase chamber → washing chamber II → oil-phase chamber → amplification chamber) and ultimately migrate into the amplification chamber (Supplementary Video S2). Subsequently, the chip is placed into a portable thermal cycler equipped with multiplex fluorescence signal reader, and was incubated at 94 °C for 1 min, followed by 45 cycles of 10 s at 95 °C, 30 s at 60 °C.

## 3 Results and discussion

### 3.1 Workflow of the USBB-MBDA system

**FIGURE 1:**
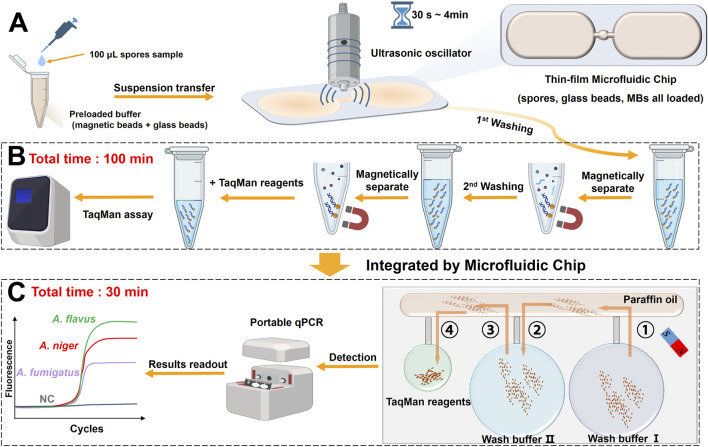
**(A)** Schematic illustration of the ultrasonic bead-beating (USBB) lysis system for fungal cell disruption. **(B)** Off-chip MBDA detection workflow based on conventional nucleic acid extraction followed by qPCR amplification. **(C)** On-chip integrated MBDA detection workflow combining MBs–based nucleic acid extraction and with MBs qPCR amplification without elution, enabling direct amplification from USBB–lysed samples.

As shown in Figure 1, the USBB-MBDA system is essentially the integration of the USBB lysis module with the MBDA detection module.

In the lysis step, a thin-film microfluidic chip containing lysis buffer, glass beads, MBs, and the target *Aspergillus* spores was positioned on the ultrasonic oscillator. Following this, the combined lysis of *Aspergillus* spores by ultrasonication and beads beating released nucleic acids, which were simultaneously captured by MBs. The MBs were subsequently transferred to the microfluidic chip with integrated washing and amplification capabilities, through external magnetic control, MBs underwent two wash steps, and were ultimately utilized for direct amplification without elution. Detailed information on thin-film and microfluidic chips is given in Supplementary Figure S10.

### 3.2 Assessment of Taqman assay

We designed three primer-probe sets targeting *A. flavus*, *A. niger*, and *A. fumigatus*, along with a fourth set specific to *C. neoformans*, using Beacon Designer 8 software (Premier Biosoft, Palo Alto, CA, United States). The three *Aspergillus*-specific primer-probe sets were engineered for triplex detection.

To validate specificity, each primer-probe set underwent cross-reactivity testing against the four target fungi (*A. flavus*, *A. niger*, *A. fumigatus*, and *C. neoformans*) and eight common respiratory and gastrointestinal pathogens (*Streptococcus pneumoniae*, *Haemophilus influenzae*, *Shigella Castellani*, *Acinetobacter baumannii*, *Escherichia coli*, *Klebsiella pneumoniae*, *Mycoplasma pneumoniae*, and *Legionella* spp.). As shown in Supplementary Figure S1, all four primer-probe sets demonstrated specific amplification under their respective target conditions, with no false-positive signals in cross-reactivity assays, confirming high specificity.

**FIGURE 2:**
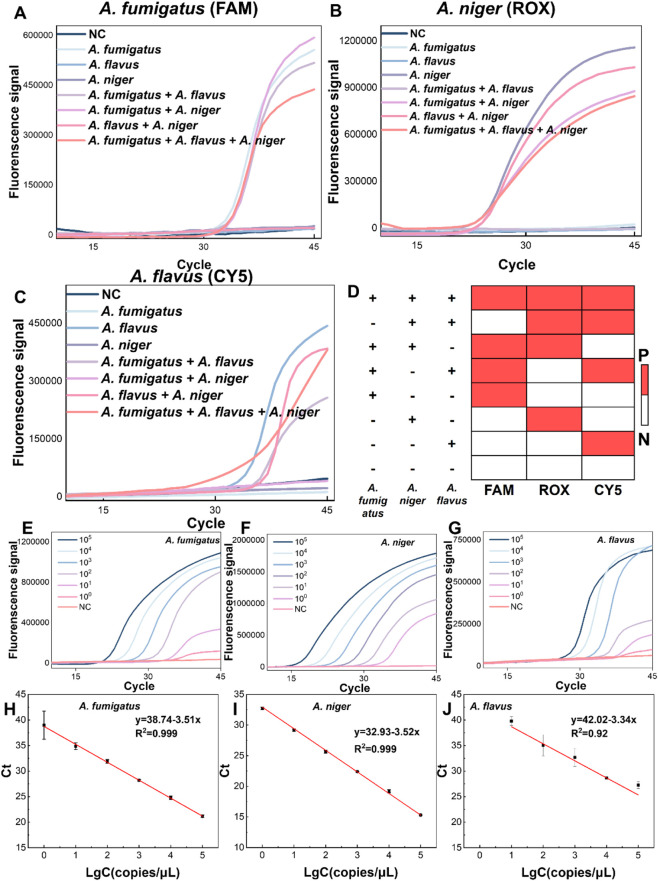
Specificity and sensitivity of the *Aspergillus*-specific triplex TaqMan assay. **(A–C)** Fluorescence–cycle curves of the triplex TaqMan assay for **(A)** *A. fumigatus*, **(B)** *A. niger*, and **(C)** *A. flavus* at a concentration of 1 × 10^3^ copies per reaction. **(D)** Heatmap summarizing the specificity evaluation of the triplex TaqMan probes based on the fluorescence–cycle curves shown in **(A–C)**. **(E–G)** Fluorescence–cycle curves of the triplex TaqMan assay for **(E)** *A. fumigatus*, **(F)** *A. niger*, and **(G)** *A. flavus* with template concentrations ranging from 1 × 10^5^ to 0 (NC) copies/μL. **(H–J)** Standard curves showing the linear relationship between Ct (threshold cycle) and lg C (template concentration) corresponding to the fluorescence–cycle curves in **(E–G)** for **(H)** *A. fumigatus*, **(I)** *A. niger*, and **(J)** *A. flavus*. Ultrapure water was used as the negative control. The specific probes were labeled with FAM, ROX, and Cy5 for identification of *A. fumigatus*, *A. niger*, and *A. flavus*, respectively. Data represent the mean values (n = 3), and error bars indicate standard deviations from triplicate measurements.3.3. Evaluation of MBDA.

Multiplex detection improves assay efficiency by enabling simultaneous analysis of multiple targets in a single reaction, achieved via probes labeled with distinct fluorescent reporters to distinguish target signals. Non-specific amplification (between primers or primer and non-specific sequences) causes false positives, and high amplification efficiency targets may suppress others, reducing sensitivity or causing false negatives. To evaluate the multiplex capability of our *Aspergillus* primer-probe sets (for *A. flavus*, *A. fumigatus*, and *A. niger*), we incorporated them into a TaqMan reaction system at a 0.75: 1: 1.5 ratio (0.75 for *A. niger,* 1 for *A. fumigatus* and 1.5 for *A. flavus,* 1 represented 0.2 μM probe and 0.4 μM primer). The system was tested against genomic DNA from single-species (*A. niger*, *A. fumigatus*, *A. flavus*), dual-species combinations (*A. niger* + *A. fumigatus*, *A. niger* + *A. flavus*, *A. fumigatus* + *A. flavus*), and a tri-species mixture (*A. niger* + *A. fumigatus* + *A. flavus*). Each genomic concentration is 10^3^ copies/μL. As shown in Figures 2A–D, fluorescence signals were exclusively detected in reactions containing their respective targets, confirming primer-probe specificity and absence of cross-reactivity.

To assess sensitivity, fungal genomic DNA extracted via standardized protocols was serially diluted from 10^5^ to 1 copies/μL and analyzed using real-time qPCR. Supplementary Figures S2A–C illustrate that the primer-probe sets for *A. flavus*, *A. niger*, and *A. fumigatus* achieved detection sensitivities down to 1 copy/μL, with excellent linear correlation (*R* ^2^ > 0.99) between Ct values and log-transformed concentrations (Supplementary Figures S2D-F). Meanwhile, sensitivity validation of the triplex TaqMan assay maintained high analytical performance: *A. fumigatus* and *A. niger* maintained a LOD of 1 copy/μL (Figures 2E,F), while *A. flavus* exhibited an LOD of 10 copies/μL (Figure 2G). Importantly, Ct values showed strong linear correlations (*R* ^2^ = 0.92 for *A. flavus*, R^2^ > 0.99 for *A. fumigatus* and *A. niger*) with log-transformed concentrations across all targets (Figures 2H–J). Supplementary Figure S3A shows that the *C. neoformans*-specific primer-probe set achieved a LOD of 10 copies/μL, also exhibiting a strong linear relationship (*R* ^2^ > 0.99) (Supplementary Figure S3B). These findings collectively demonstrate that our triplex *Aspergillus* primer-probe system achieves exceptional specificity and sensitivity, enabling accurate detection of *A. flavus*, *A. fumigatus* and *A. niger* within a broad concentration range in mixed samples.

### 3.3 Evaluation of MBDA

Conventional MB-based protocols generally employ low-salt alkaline reagents (e.g., ddH_2_O, TE Buffer, TB Buffer) for MBs elution. However, only a small fraction of nucleic acid-containing eluate is transferred to subsequent amplification reactions, leading to increased operational workflow complexity, reduced target concentration in amplification systems, and potential impairment of the LOD. To address this, our study sought to directly introduce MBs with adsorbed nucleic acids into amplification, thereby enabling full target participation and improving detection sensitivity.

**FIGURE 3:**
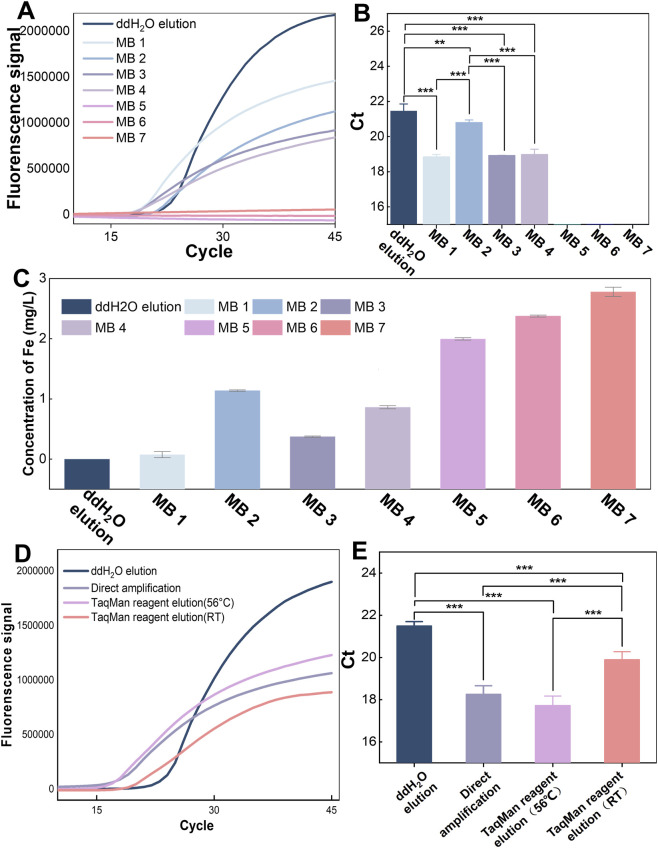
Optimization of the MBDA. (1) Comparison of magnetic beads for on-bead amplification. **(A)** Fluorescence–cycle curves of direct qPCR amplification after nucleic acid extraction from *A. niger* spores using seven magnetic bead types. **(B)** Ct value comparison of different magnetic beads.**(C)** Residual iron ion concentrations in the qPCR system after amplification with different magnetic beads, quantified by atomic absorption spectroscopy. (2) Evaluation of magnetic bead elution conditions. **(D)** Fluorescence–cycle curves obtained under different elution conditions. **(E)** Ct value comparison under different elution conditions. *A. niger* spores (1 × 10^5^ spores per reaction) were used as the target. Data represent mean values (n = 3); error bars indicate standard deviations. Statistical differences were analyzed by one-way ANOVA and Student’s t-test: *p < 0.05, **p < 0.01, ***p < 0.001.

In this study, seven commercial magnetic beads (MB 1–MB 7) were systematically evaluated for their compatibility with direct amplification. MB 1, MB 2 and MB 5 were silicon-based magnetic beads, MB 3 and MB 6 were silica hydroxyl magnetic beads, while no information on the bead types of MB 4 and MB 7 was available in public datasets. Notably, MB 2 and MB 3 possessed a protective interlayer between Fe_3_O_4_ cores and silica outer layer. As shown in Figures 3A,B, MB 1-MB 4 successfully supported direct amplification, whereas structurally analogous MB 5 (silicon-based) and MB 6 (silica hydroxyl) failed, indicating that the surface modification type of magnetic beads is not the critical determinant affecting the feasibility of direct amplification. Hapsianto et al. previously achieved direct amplification using gold-coated beads (Hapsianto et al., 2022), while literature reports suggest qPCR inhibition by 1 mg/L heavy metal ions (Chen and Chang, 2012; Combs et al., 2015). To investigate the impact of Fe ions released from MBs, atomic absorption spectroscopy was employed to quantify the Fe concentration in the post-thermal cycling PCR mixture. As shown in Figure 3C, amplification-compatible MBs (MB 1–MB 4) released significantly less Fe during thermal cycling than non-compatible ones (MB 5–MB 7) Notably, MBs(MB1, MB 3 and MB 4) releasing Fe ions at 75–860 ng/mL allowed high-efficiency direct amplification. MB 2’s delayed Ct values suggested partial inhibition despite successful amplification, likely due to relatively high iron concentration (1,375 ng/mL). In contrast, MBs with >2000 ng/mL iron completely inhibited amplification (Figures 3A–C). This demonstrates that maintaining magnetic beads stability during thermal cycling to prevent Fe ion leaching into the reaction mixture is a critical determinant for achieving direct amplification.

We systematically compared four nucleic acid processing strategies: (1) direct amplification with magnetic beads, (2) 56 °C incubation followed by beads removal, (3) room-temperature (RT) incubation followed by beads removal, and (4) conventional 100 μL ddH_2_O elution (only 10 μL was used for amplification) (Figures 3D,E). Notably, direct amplification and 56 °C incubation protocols showed similar Ct values, while RT incubation achieved earlier Ct detection than conventional elution. MBs sedimentation in solution impaired the ABI7500’s fluorescent signal detection, resulting in relatively lower fluorescence intensity for direct amplification compared to ddH_2_O elution. These results demonstrated that although direct amplification with MBs may introduce potential inhibition, this strategy yields higher detection sensitivity by enabling full utilization of the total template volume, as opposed to the conventional method that only incorporates a 10 μL aliquot of the eluted template. Consequently, direct amplification confers a significant enrichment effect, increasing the template concentration by approximately 10-fold and reducing the Ct value by roughly 3 cycles. In addition, MBs removal prior to amplification requires at least 5–10 min of vortex mixing to ensure high nucleic acid elution efficiency, this additional step not only elevates operational complexity but also significantly prolongs the overall detection time. Thus, the direct amplification protocol achieves an optimal balance between amplification efficiency and procedural simplicity.

### 3.4 Verification of USBB-MBDA system

**FIGURE 4:**
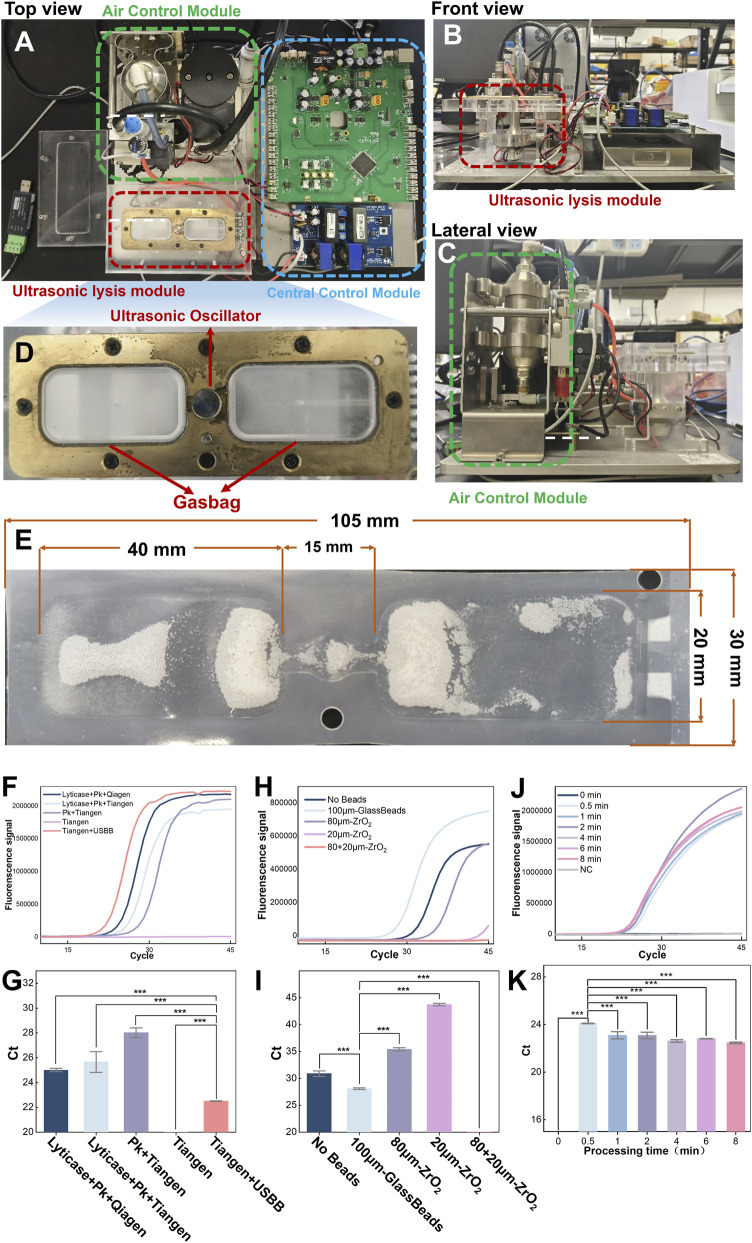
Schematic illustration and validation of the USBB system. **(A)** Top view, **(B)** front perspective view, and **(C)** lateral view of the USBB device. **(D)** Enlarged view of the ultrasonic lysis module highlighted in **(A)**. **(E)** Thin-film microfluidic chip designed for sample loading during ultrasonication. **(F)** Fluorescence–cycle curves showing USBB-mediated enhancement of lysis efficiency compared with different DNA extraction methods. **(G)** Ct value comparison of different DNA extraction methods. **(H)** Fluorescence–cycle curves obtained using different bead types for USBB-assisted lysis. **(I)** Ct value comparison of different bead types. **(J)** Fluorescence–cycle curves obtained at different sonication processing times. **(K)** Ct value comparison of different sonication processing times. *A. niger* spores (1 × 10^5^ spores per reaction) were used as the detection target. Data represent mean values (n = 3); error bars indicate standard deviations. Statistical differences were analyzed by one-way ANOVA and Student’s t-test: *p < 0.05, **p < 0.01, ***p < 0.001.

The USBB-MBDA method was developed as a rapid, sensitive, and user-friendly detection protocol specifically optimized for fungal spores with rigid cell walls, and it incorporates ultrasonic-assisted mixing as a key preprocessing step. As shown in Supplementary Video S1, methyl red solution and TE buffer achieved complete mixing within 10 s, forming a homogeneous yellow solution; this ensures the uniform distribution of fungal spores in the reaction system, sufficient exposure to ultrasonication for effective lysis, and improved overall spore lysis efficiency. Compared with other lysis approaches, USBB demonstrates outstanding lytic performance (Figures 4F,G). When compared with the combined use of Qiagen commercial nucleic acid extraction kit and lyticase treatment, as recommended by the manufacturer, USBB achieves 85.5% of the extraction efficiency obtained by the reference method (Supplementary Figure S8). Despite differences in lysis performance, USBB offers advantages in processing speed and system integration. This modification eliminated the need for a prolonged constant-temperature incubation step using Proteinase K (Pk), which typically requires 30 min to 2 h, thereby substantially reducing the total extraction time. As shown in Figures 4H,I, glass beads were identified as the most effective bead type. Furthermore, just 1 min of ultrasonic treatment was sufficient to achieve relatively high DNA release efficiency (Figures 4J,K).

**FIGURE 5:**
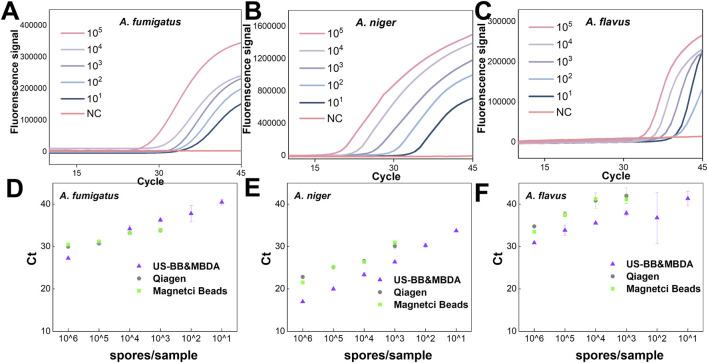
Detection sensitivity of the USBB–MBDA method. **(A–C)** Sensitivity evaluation of USBB–MBDA for the detection of **(A)** *A. fumigatus*, **(B)** *A. niger*, and **(C)** *A. flavus* at different spore concentrations. **(D–F)** Comparison of amplification performance obtained from nucleic acid extracts prepared using three different extraction methods for **(D)** *A. fumigatus*, **(E)** *A. niger*, and **(F)** *A. flavus* over a concentration range of 10^1^–10^6^ spores/sample.

In comparative experiments against two commercial DNA extraction kits, we evaluated the sensitivity of all three methods using *A. flavus*, *A. fumigatus*, and *A. niger* as target species across a concentration range of 10^5^ to 10 spores/test, Results were illustrated in Figure 5. Extensive validation showed the primer-probe sets have excellent specificity and sensitivity, with no cross-reactivity or false positives. Given this, all Ct values within 45 cycles can be regarded as positive, and USBB-MBDA stably detected targets at 10 spores/test (100 spores/mL). (Figures 5A–C), achieving a 100-fold improvement in detection limit compared to commercial kits (10^3^ spores/test, equivalent to 10^4^ spores/mL) (Figures 5D–F). By eliminating prolonged isothermal enzymatic lysis and labor-intensive centrifugation steps, USBB-MBDA further demonstrated cost-effectiveness, streamlined workflow, and significantly shorter processing time. A comprehensive comparison is detailed in Supplementary Table S2. Meanwhile, USBB-MBDA achieved reliable detection of *C. neoformans* with hyperthickened capsules at the LOD of 100 spores/test (equivalent to 10^3^ spores/mL), as shown in Supplementary Figure S4.

The competitive sensitivity assay (Supplementary Figure S5; Supplementary Table S3) confirmed that the USBB-MBDA system could reliably detect *A. fumigatus*, *A. flavus*, and *A. niger*at concentrations as low as 10 spores per test, despite high background interference (10^4^ spores/test) from the other two species. These findings underscore the robustness of the system for the simultaneous detection of the three *Aspergillus* species.

To evaluate the overall performance of the USBB-MBDA system, we compared it with other reported studies, as shown in Table 1. Compared with existing studies and related methods, the USBB-MBDA system exhibits certain advantages in fungal lysis efficiency, LOD, stable clinical performance, turnaround time, cost-effectiveness, and user-friendliness. When compared with the bead-beating-based BIOFIRE® FILMARRAY® 2.0 System (bioMérieux, Marcy-l'Étoile, France), the USBB system incorporates an ultrasonic design. This modification theoretically enhances cell lysis efficiency but may cause genomic DNA fragmentation under certain conditions, impairing downstream molecular detection. Figures 4H,I showed that all ZrO_2_ bead sizes inhibited amplification, whereas glass beads enhanced amplification efficiency versus no-beads controls, despite similar nucleic acid yields (ZrO_2_ beads: 3.77 ng/μL; glass beads: 3.63 ng/μL; no beads: 0.84 ng/μL). A meta-analysis (Tansarli and Chapin, 2020) of the BIOFIRE^®^ FILMARRAY^®^ Meningitis/Encephalitis (ME) Panel (FA ME panel) (bioMérieux, Marcy-l'Étoile, France) reported an overall sensitivity of 90% (95% CI: 86%–93%) and specificity of 97% (95% CI: 94%–99%). However, a relatively high false-negative rate (20%) was observed for *C. neoformans* (Lee et al, 2019; Ramanan et al, 2018). As an established diagnostic platform, the FAME panel demonstrates an LOD ranging from 100 to 1000 CFU/mL for its target pathogens (Evaluation, 2022). However, its high per-test cost (>$100 per panel) (Tansarli and Chapin, 2020)imposes a significant financial burden on patients. In contrast, the USBB-MBDA system achieved an LOD of 10 spores/test for *Aspergillus* and 100 spores/test for *C. neoformans* in this study, with a per-test cost of approximately $1. This approach not only meets clinical needs for early detection but also substantially reduces the economic burden on patients.

**TABLE 1:** Comparison of performance parameters of USBB-MBDA and existing fungal detection methods.

Study/Method	Target pathogen	Lysis	Detection	LOD	Clinical performance (Sens/Spec)	Time-to-result	Cost per test	Ref.
USBB-MBDA	*A. Fumigatus* *A. flavus* *A. niger* *C. neoformans*	Ultrasonication Beads beating	qPCR with magnetic beads	*Aspergillus* 10 spores/test *C. neoformans:* 100 spores/test	Sens: 100% (95% CI: 87.2–100.0) Spec: 100% (95% CI: 69.2–100.0)	35 min	$1	This study
FA-ME panel	*C. neoformans*	Beads beating	nested PCR (nPCR)	NR	Sens: 90% (95% CI: 86%–93%) Spec: 97% (95% CI: 94%–99%)	60 min	$100	Tansarli and Chapin (2020)
RCCD	*P. jirovecii*	Chemical lysis (TIANGEN)	RPA-CRISPR/Cas12	1 copy/reaction	Sens: 100% (39/39)	45 min	NR	Wu et al. (2025)
LMC	*A. Fumigatus*	Chelex-100; beads beating	LAMP	4 × 10^4^ spores/sample	NR	80 min	$0.7	Lu et al. (2022)
EL-ECD	*C. neoformans*; *C. gattii*	Electroporation lysis	Electrochemical detection	60–100 pg/mL	NR	NR	NR	Kong et al. (2024)
TOEC	*C. albicans*	Enzymatic lysis (TOLO)	ERA-CRISPR/Cas12a	100 ag/μL	NR	∼50 min	NR	Zeng et al. (2025)
(CA-OL-CDA)	*C. albicans*	Chemical lysis (TIANGEN)	CDA and HNB endpoint visual judgement	6.2 fg/mL	Sens: 100% (95% CI: 97.5–100.0) Spec: 100% (95% CI: 98.7–100.0)	>90 min	NR	Zhang et al. (2025)

Abbreviations: NR, not reported; Sens/Spec, clinical sensitivity/specificity; BALF, bronchoalveolar lavage fluid; *P. jirovecii, Pneumocystis jirovecii; C. gattii, Cryptococcus gattii; C. albicans, Candida albicans;* CDA, Closed dumbbell-mediated isothermal amplification; HNB, hydroxy naphthol blue.

### 3.5 Feasibility of the microfluidic chip

**FIGURE 6:**
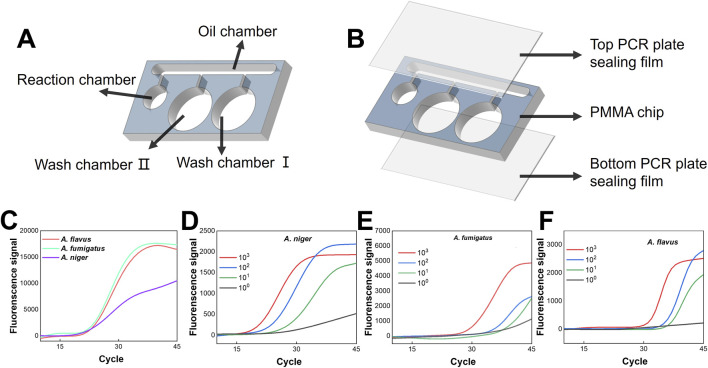
On-chip implementation and sensitivity evaluation of the USBB–MBDA method. **(A)** Schematic illustration of the microfluidic chip. **(B)** Assembly of the microfluidic chip with a three-layer composite structure. **(C)** On-chip triplex detection of *Aspergillus* species using genomic DNA standards spiked at a concentration of 10^4^ copies reaction^−1^. **(D–F)** Sensitivity evaluation of the USBB–MBDA method implemented on the microfluidic chip using serially diluted spores of **(D)** *A. niger*, **(E)** *A. fumigatus*, and **(F)** *A. flavus*, with concentrations ranging from 10^3^ to 1 spore/test.

To integrate magnetic bead washing and amplification steps, we developed a microfluidic chip (structural details shown in Figure 6A). During operation, TaqMan mix, Washbuffer I, and Washbuffer II are sequentially loaded into designated chambers, followed by liquid paraffin injection into the oil-phase chamber to establish phase separation. Guided by an external magnet, magnetic beads in Washbuffer I move through successive chambers and ultimately positioned in the amplification chamber, where they undergo thermal cycling for direct amplification. The thermal cycle and fluorescence detection equipment is shown in Supplementary Figure S6. Experimental validation confirmed that the liquid interfaces within the chip remained intact with no detectable intermixing following thermal cycling, thereby effectively maintaining physical isolation between chambers. The on-chip detection system exhibited precise target-specific recognition, confirming the robust functionality of the pre-established triplex detection assay (Figure 6C). Specifically, amplification curves displayed reduced conformity to the typical sigmoidal profile relative to those from mature commercial instruments (e.g., ABI7500). This discrepancy was due to the employment of a self-built PCR prototype rather than a fully optimized commercial system. Supplementary Figure S7 showed pre- and post-amplification fluorescence intensity across three signal channels, with ΔMGV confirming effective amplification of all three targets. Overall, despite being a self-built prototype, the on-chip system achieved reliable target detection, validating the applicability of the triplex assay. Although the MBs successively traversed the aqueous-oil interfaces during their translocation within the chip, resulting in a beads loss of 14.36% ± 2.46% (Supplementary Figure S9), Figures 6D–F demonstrates that the USBB-MBDA protocol implemented on the integrated microfluidic chip maintained high detection sensitivity (10 spores/test) for three *Aspergillus* species despite combining nucleic acid purification and amplification steps. This integrated system successfully unified washing and amplification workflows while preserving multiplex detection capabilities without sensitivity loss. Importantly, the magnet-guided beads transport mechanism exhibits full automation potential through programmable magnetic field control.

### 3.6 Validation of USBB-MBDA using clinical samples

**FIGURE 7:**
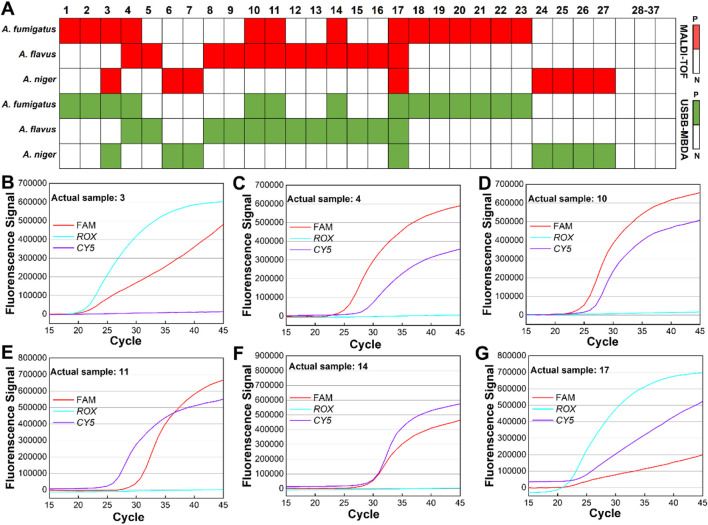
Clinical validation of the USBB–MBDA method. **(A)** Heatmap summarizing the detection results obtained from clinical samples. **(B–G)** Triplex real-time fluorescence amplification curves of 6 multiple infection samples from 27 positive clinical samples.

To evaluate the clinical applicability and reliability of the USBB-MBDA system, a total of 37 clinical samples were collected, with their diagnostic statuses pre-confirmed by MALDI-TOF, yielding 27 positive and 10 negative samples. The results of the USBB-MBDA system were consistent with those of MALDI-TOF in identifying all positive samples, and no false-positive results were detected among the 10 negative samples (Figure 7A). These findings demonstrated that the USBB-MBDA system achieved a sensitivity of 100% (95% CI: 87.2–100.0) and a specificity of 100% (95% CI: 69.2–100.0) (Table 2), confirming excellent diagnostic accuracy. Furthermore, the multiplex detection capability of the USBB-MBDA system was validated using samples with mixed Aspergillus infections, which further corroborated its reliability and suitability for detecting complex clinical fungal infections (Figures 7B–G). All positive detection results are presented in Supplementary Figure S11.

**TABLE 2:** Sensitivity and specificity analysis of USBB-MBDA.

Methods	Positive		Negative		Total tested (n)	Key clinical performance metrics	
	TP	FP	TN	FN		Sensitivity (95% CI)	Specificity (95% CI)
USBB-MBDA	27	0	10	0	37	100.0 (87.2–100.0)	100.0 (87.2–100.0)
MALDI-TOF	27	0	10	0	37	—	—

Abbreviations: TP, true positive; FP, false positive; TN, true negative; FN, false negative.

## 4 Conclusion

In this study, an integrated ultrasonication-based beads-beating and magnetic beads–based direct amplification system (USBB–MBDA) was established for rapid fungal nucleic acid extraction and detection. Upon integration into a flexible thin-film microfluidic chip, the system completed the entire workflow from sample to result within 35 min, including 5 min for fungal DNA release and 30 min for amplification. The microfluidic chip achieved an 85.5% lysis efficiency for *Aspergillus* at a concentration of 10^5^ spores per sample. Magnetic beads releasing Fe ions in the range of 75–860 ng/mL did not exert significant inhibition on PCR reactions, confirming their compatibility with amplification. Complete transfer of the nucleic acid extract into subsequent amplification resulted in a 100-fold improvement in detection sensitivity. Using this system, simultaneous triplex detection of *Aspergillus fumigatus*, *Aspergillus flavus*, and *Aspergillus niger* was achieved with a limit of detection as low as 10 spores per test. Besides, USBB–MBDA system exhibited 100% (95% CI: 87.2–100.0) sensitivity and 100% (95% CI: 69.2–100.0) specificity in clinical validation, confirming its reliable analytical performance and potential for clinical *Aspergillus* detection.

In addition to *Aspergillus* species, USBB–MBDA was also effective for detecting *C*. *neoformans*, despite its exceptionally thick capsule. These results suggest that the system is suitable for pathogens with structurally robust cell walls. Our study establishes an integrated nucleic acid extraction and magnetic bead–enabled direct amplification strategy that provides a versatile approach for the development of automated analytical platforms, with broad applicability to *in vitro* and pathogen diagnostic applications.

## Data Availability

The original contributions presented in the study are included in the article/Supplementary Material, further inquiries can be directed to the corresponding authors.
