# Supplementary material for: Multiplex on-chip detection of Aspergillus by integrated ultrasonication-based bead beating lysis and magnetic beads direct amplification
Source: Front Bioeng Biotechnol. 2026 Feb 19;14:1775828. doi: 10.3389/fbioe.2026.1775828 (PMC12960495; doi:10.3389/fbioe.2026.1775828)
Supplement: Supplementary file 1 [file Supplementaryfile1.docx]

**Supporting Information**

**Multiplex on-chip detection of *Aspergillus* by integrated ultrasonication-based bead beating lysis and magnetic beads direct amplification**

Jinyu Zhong^1†^, Shiliang Zhang^3†^, Wei Huang^1†^, Jie Cheng^1^, Xiaoning Li^4^, Sen Wang^5^, Tong Zhang^2,6^*, Guodong Sui^1,7^**

^1^ Shanghai Key Laboratory of Atmospheric Particle Pollution and Prevention (LAP3), Department of Environmental Science and Engineering, Fudan University, Shanghai, China

^2^ Shanghai Sci-Tech Inno Center for Infection & Immunity, Shanghai, China

^3^ Department of Clinical Laboratory, The Fifth People's Hospital of Wuxi, Wuxi, China

^4^ IngeDx Technologies Co., Ltd, Suzhou, China

^5^ Department of Infectious Diseases, Shanghai Key Laboratory of Infectious Diseases and Biosafety Emergency Response, National Medical Center for Infectious Diseases, Huashan Hospital, Shanghai Medical College, Fudan University, Shanghai, China

^6^ Department of Clinical Laboratory, Shanghai East Hospital, Shanghai, China

Shanghai 200120, P. R. China

^7^ Shanghai Institute of Infectious Disease and Biosecurity, Fudan University, Shanghai, China

^†^ These authors contributed equally to this work.

^*^Corresponding author. ^**^ Corresponding author.

E-mail address: [shmzhyzt@163.com](mailto:shmzhyzt@163.com) (T. Zhang), [gsui@fudan.edu.cn](mailto:gsui@fudan.edu.cn) (G.D. Sui)

Content

[Table. S1. Nucleic acid sequences involved in this study 6](#_Toc220891269)

[Table. S2. Comparison of three nucleic acid yield methods. 8](#_Toc220891270)

[Table. S3.Competitive sensitivity assay of USBB&MBDA for detecting three *Aspergillus* Species 9](#_Toc220891271)

[**Fig. S1**. Heat map of cross-reactivity experiments among four specific primer-probe 9](#_Toc220891272)

[**Fig. S2.** Sensitivit of primer-probe sets for three *Aspergillus* species. 10](#_Toc220891273)

[**Fig. S3.** Sensitivit of C. neoformans-specific primer-probe set. 10](#_Toc220891274)

[**Fig. S4.** Detection sensitivity of US-BB&MBDA for C. neoforams 11](#_Toc220891275)

[**Fig. S5.** Competitive sensitivity assay of USBB&MBDA for detecting 11](#_Toc220891276)

[**Fig. S6.** Image of (A) thermal cycle module，(B) fluorescence detection module and (C) chip working on the equipment. 12](#_Toc220891277)

[**Fig. S7.** Fluorescence Comparison Images of On-Chip Multiplex TaqMan Assays Before and After Amplification. Δ MGV denotes the difference in the images’ mean gray value before and after PCR. 12](#_Toc220891278)

[**Fig. S8.** Ct–concentration linearity for *A. niger* at different concentrations using two extraction methods. (A) Qiagen; (B) USBB&MBDA 13](#_Toc220891280)

[**Fig. S9.** Loss of magnetic beads during traversal across oil-water interface in chip 14](#_Toc220891286)

[**Fig. S10.** Schematic diagram of the structure and parameters of the thin-film chip (A) and microfluidic chip (B). 15](#_Toc220891293)

[**Fig. S11.** Detection results of 27 positive actual samples 16](#_Toc220891295)

Table. S1. Nucleic acid sequences involved in this study

| Target | Primer＆Probe | Sequence（5’-3’） | Target sequence（5’-3’） |
| --- | --- | --- | --- |
| *A. niger* | Forward | ATTACCGAGTGCGGGTCCTT | GTAGGTGAACCTGCGGAAGGATCATTACCGAGTGCGGGTCCTTTGGGCCCAACCTCCCATCCGTGTCTATTGTACCCTGTTGCTTCGGCGGGCCCGCCGCTTGTCGGCCGCCGGGGGGGCGCCTCTGCCCCCCGGGCCCGTGCCCGCCGGAGACCCCAACACGAACACTGTCTGAAAGCGTGCAGTCTGAGTTGATTGAATGCAATCAGTTAAAACTTTCAACAATGGATCTCTTGGTTCCGGCATCGATGAAGAACGCAGCGAAATGCGATAACTAATGTGAATTGCAGAATTCAGTGAATCATCGAGTCTTTGAACGCACATTGCGCCCCCTGGTATTCCGGGGGGCATGCCTGTCCGAGCGTCATTGCTGCCCTCAAGCCCGGCTTGTGTGTTGGGTCGCCGTCCCCCTCTCCGGGGGGACGGGCCCGAAAGGCAGCGGCGGCACCGCGTCCGATCCTCGAGCGTATGGGGCTTTGTCACATGCTCTGTAGGATTGGCCGGCGCCTGCCGACGTTTTCCAACCATTCTTTCCAGGTTGACCTCGGATCA |
|  | Reverse | GCATTTCGCTGCGTTCTTCAT |  |
|  | Probe | ROX-CTATTGTACCCTGTTGCTTCGGCGGGC-BHQ2 |  |
| *A. fumigatus* | Forward | CTTTGGTTTCTGTTGCCTG | AACTCATCTCTGTGAGAGGAATGAACTTTGGTTTCTGTTGCCTGTCCAAGGCTTAATCTCGTCAGTGCTCATTGCTACCGTCCTCCCAATCCACATGTTTGGTGCGAAAGAGACTCGGACCTTTCTCGCCTGGATTTAGAGGTTGATCCTCTATTGAGAGTTAATGCTCGGTTATACGCGCAGTTATATACGCCGAGAATCATAAGAAAAGCATAACCGTAGCACAGAGATTTGAGCCCAAAGCCCTGCAGCCTCGGCTAGATTCCGTCCCAGTCAATCCAGCCAAAGGTGTTCAAGAGTTCCACTAACGGCACTCTCTTCCCCGAATAGTTGACTAAAGAGGGCATCGAGACTAGGTTGGTGGGAAGCCAGAACCGGGGGCACATTGAGAATACGATCCAGACTCCGACTAAGGATCCT |
|  | Reverse | ATGCTTTTCTTATGATTCTCGG |  |
|  | Probe | FAM-ACGGTAGCAATGAGCACTGACGA-BHQ1 |  |
| *A. flavus* | Forward | CTCACGGTCAACTACAGC | ATGATGACCAAACTCTGGATGATCTTTGGCTGCATGGCGGCGGCCTTCTCAACCGCTACATCCACGAAACTCCTTCAAGAACAGAATGTTCCACTTACTCTCGCCCTCGAAATCGCCCAGGATGCAGTTCAGGACTGCGCCAAGAAGCAATACAGCGTATCGGCTGCTGTAGTTGACCGTGAGGGCGTCCTGCGCGCGCTCCTTCGGGCTGACAATGCAGCTATCCACACCCCCGAGGCGGCGCGCCGGAAGGCGTATACAGCTGCATCGTCGCGTACCGCGACCAGTACTATGGTCAAGAACATTCAAAATCCTGGCGCTGCCCAACTTGCCGCCGTTGATGATTTCCTAATTCTCGCTGGTGGCGTGCCAATCAAAGTCGGAAATGAGACAATCGGCGCTGTCGGTGTTGGTGGTGCGCCCAGTGGGGATTTCGATGAGGCTTGTGCGATGGTCGCACTCCAACAGGTAGCAGACAAGCTGCTGTAG |
|  | Reverse | GCTACATCCACGAAACTCC |  |
|  | Probe | CY5-ACTGCATCCTGGGCGATTTCGAG-BHQ2 |  |
| *C. neoformans* | Forward | GCTTACCTCATCTACTCCATCGG | CTCGAATGCGGGAGATCTCGACACCCTAGCCGCTTTTCAAGCCTACCTCATCTACTCCATCGGGACATACTTCTTCCCCATCCAAGGAAATCCACTTGTGGATGATGCCACAATGTTTACTTTACAGGAGATGGCTTTCCGTACGGCGCAGAGCGGATTGGTCTCCCAAGCCGAATTTGGCCGATCACGGCCAAAGTGGGAATCTTGGATTGTTGTTTCAGCAAAACGCAGGGCAATTTTCGCCTTCTACCTGCTCAGCAACGTGTATAACGCGGACAACTACGTCCCCAATTTCCTTGCGGAGGAGCTGAAAGAAGTGTATGCGCCGGATGCGAAACGGCTATGGGAGGCAAGGTCTCGTATTGATTGGGAGCACGAGTACAGTCAATATCTGTCTCAGTGGGAGGATGGACAGCTGAG |
|  | Reverse | TGATCGGTCAAATTCGGCTTG |  |
|  | Probe | HEX-TTCCTTGGATGGGGAAGAAGTA-BHQ1 |  |

Table. S2. Comparison of three nucleic acid yield methods.

|  | Qiagen | Tiangen | US-BB&MBDA |
| --- | --- | --- | --- |
| price | ~ 5 $ | ~ 3 $ | ~ 1 $ |
| enzyme | lyticase；proteinase K | lyticase；proteinase K | / |
| Time | more than 4 h (lyticase incubation ~ 30min; proteinaseK incubation ~＞2h) | more than 4 h  (lyticase incubation~ 30min; proteinaseK incubation ~＞2h) | ~ 1.5 h |
| equipment | water bath pot；  centrifuge with accurate  speed controlling | water bath pot；  shaker and  magnetic separation device | portable ultrasonication device；magnet |
| procedure | tedious, many steps of centrifugation and pipetting | tedious, many steps of magnetic attraction and pipetting. | simple, just use a magnet to pull the magnetic beads. |

Table. S3.Competitive sensitivity assay of USBB&MBDA for detecting three *Aspergillus* Species

| Species | Competitive factor | LOD (spores/test) |
| --- | --- | --- |
| *A. fumigatus* | *A. niger* (10^4^ spores/test)  *A. flavus* (10^4^ spores/test) | 10 |
| *A. flavus* | *A. niger* (10^4^ spores/test)  *A. fumigatus* (10^4^ spores/test) | 10 |
| *A. niger* | *A. flavus* (10^4^ spores/test)  *A. fumigatus* (10^4^ spores/test) | 10 |


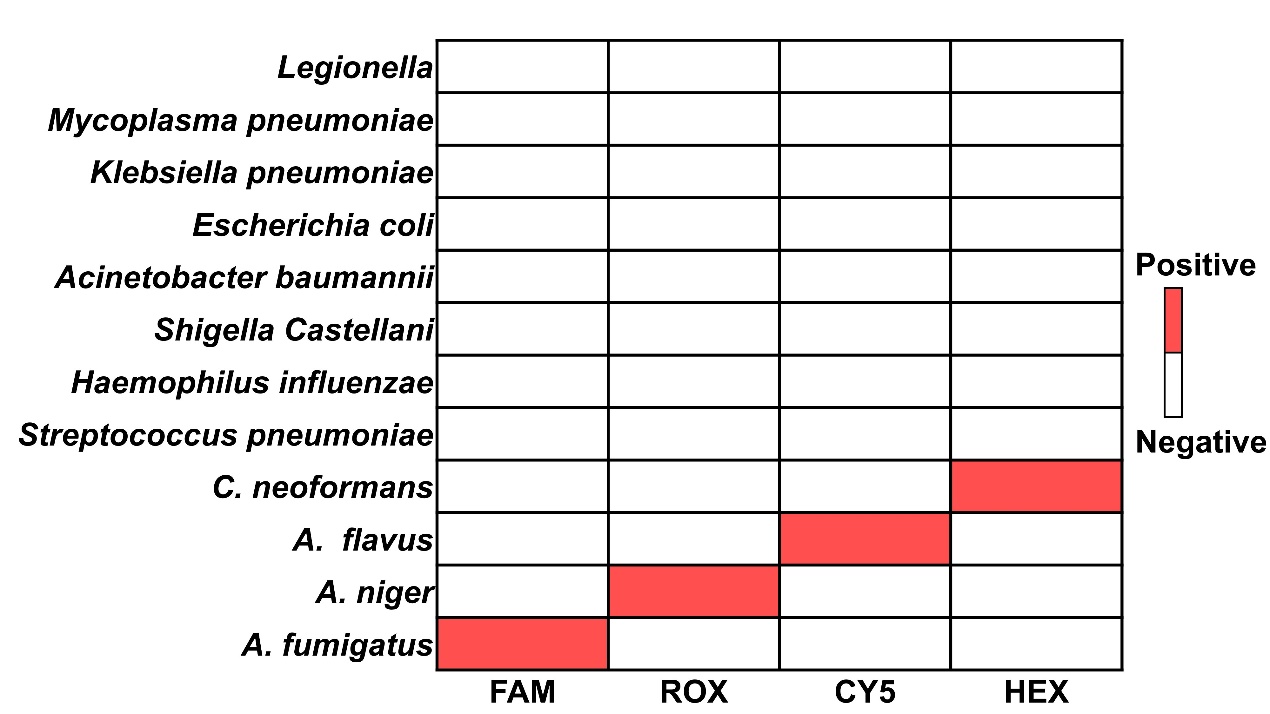


**Fig. S1**. Heat map of cross-reactivity experiments among four specific primer-probe
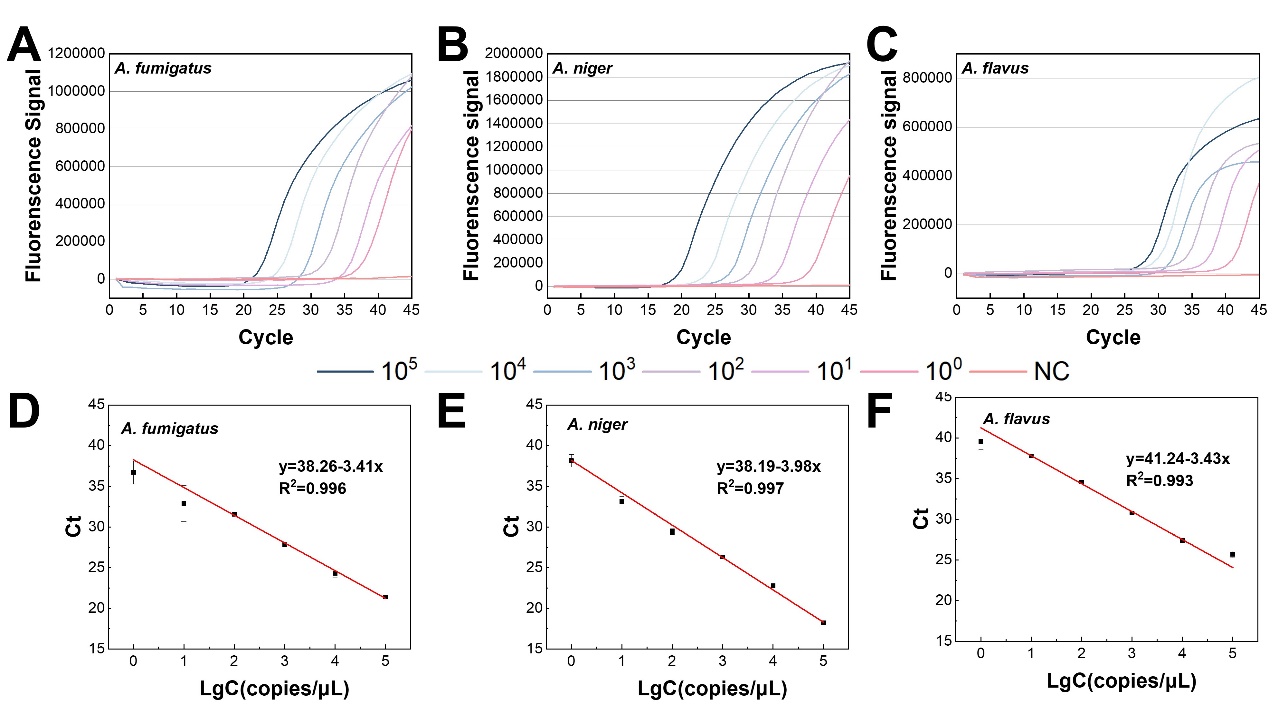
sets.

**Fig. S2.** Sensitivit of primer-probe sets for three *Aspergillus* species. Real-time fluorescence monitoring of amplification reactions of (A) *A. fumigatus*, (B) *A. niger* and (C) *A. flavus* with concentration ranging from 1 × 10^5^ to 0 (NC) copies/reaction. Ultrapure water was used as the negative control. Standard linear fitting curve of Ct (threshold cycle) vs. lg C (concentration) generated by the triplex TaqMan assay of (D) *A. fumigatus*, (E) *A. niger* and (F) *A. flavus.* Data represents the mean (n = 3). Error bars are from triplicate measurements.


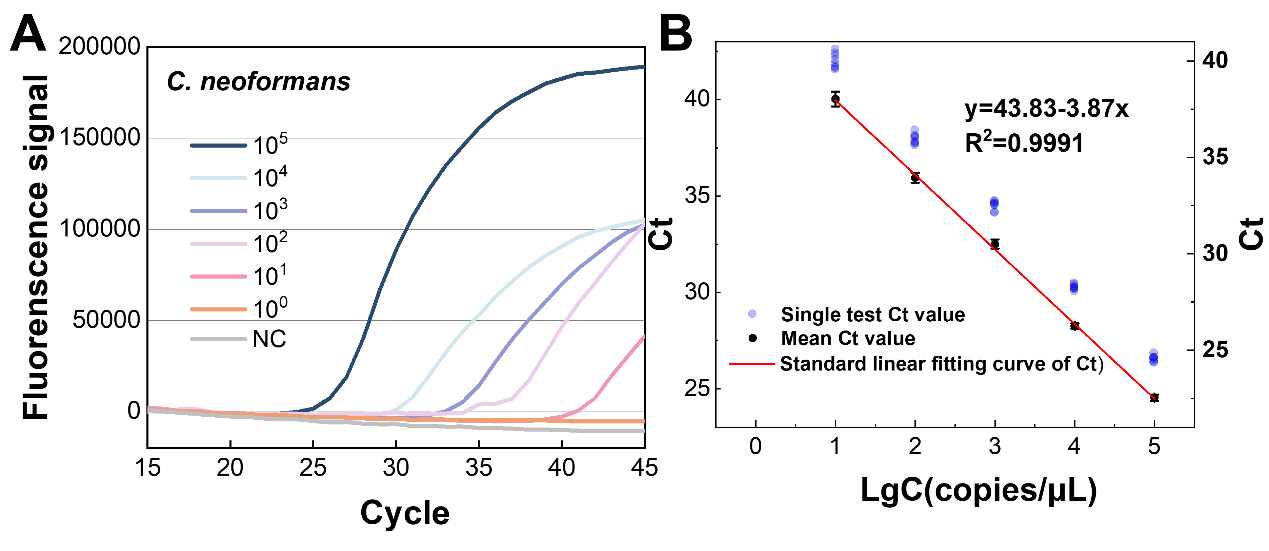


**Fig. S3.** Sensitivit of C. neoformans-specific primer-probe set. (A) Real-time fluorescence monitoring of amplification reactions of *C. neoformans* with concentration ranging from 1 × 10^5^ to 0 (NC) copies/reaction. (B) Standard linear fitting curve of Ct (threshold cycle) vs. lg C (concentration). Data represents the mean (n = 3). Error bars are from triplicate measurements.


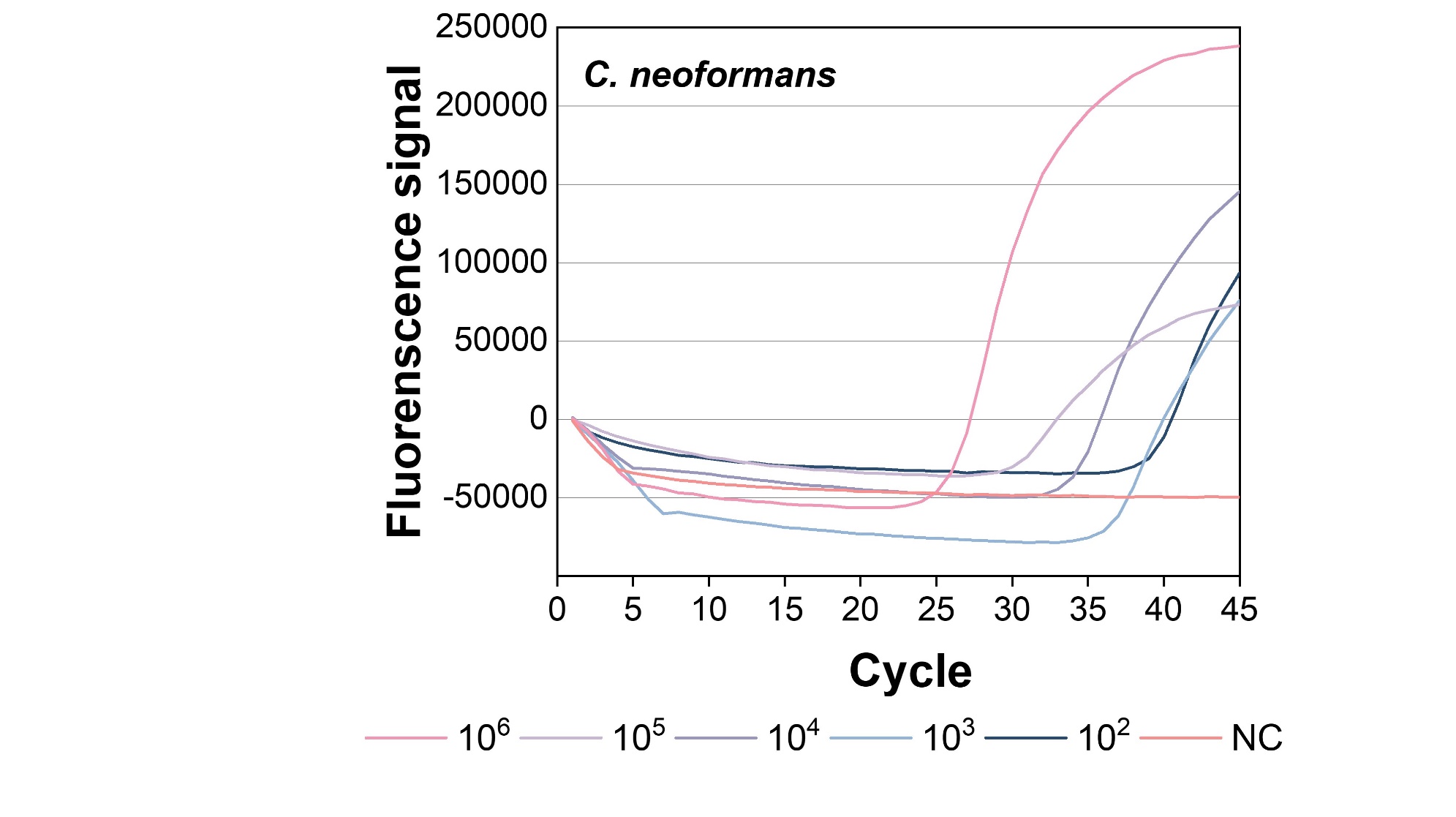


**Fig. S4.** Detection sensitivity of US-BB&MBDA for C. neoforams， with concentrations ranging from 10^6^ to 10^2^ spores/reaction，Ultrapure water was used as the negative control（NC）.


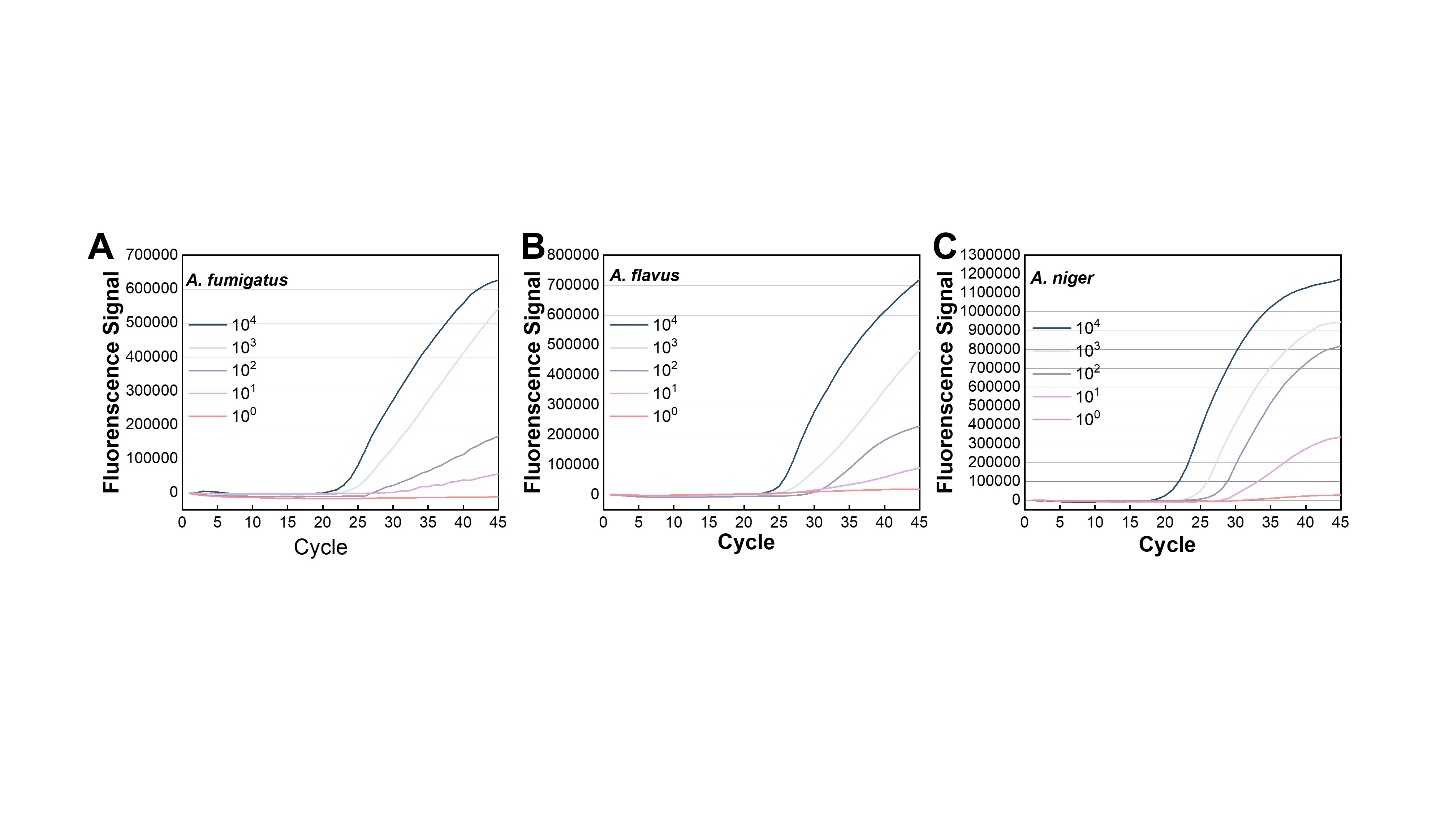


**Fig. S5.** Competitive sensitivity assay of USBB&MBDA for detecting (A) *A. fumigatus*, (B) *A. flavus* and (C) *A. niger* with concentration ranging from 1 × 10^4^ to 1 copies/reaction. Data represents the mean (n = 3).


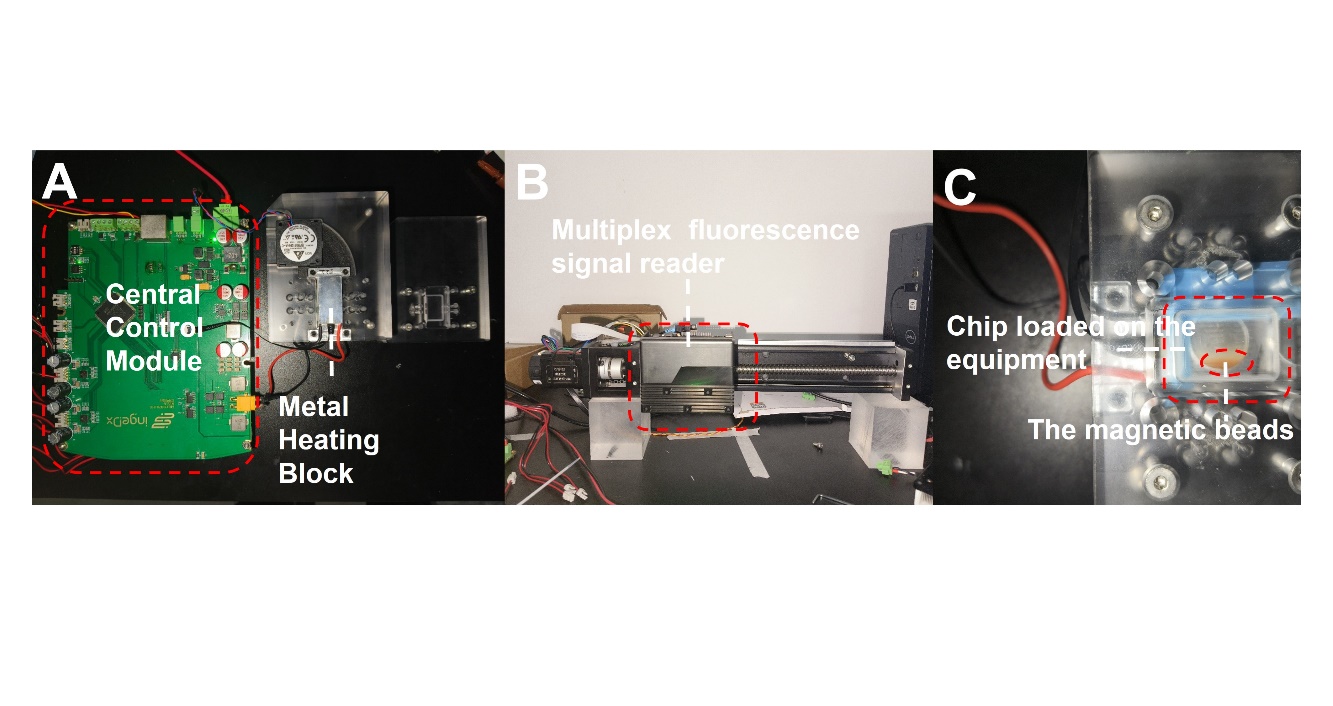


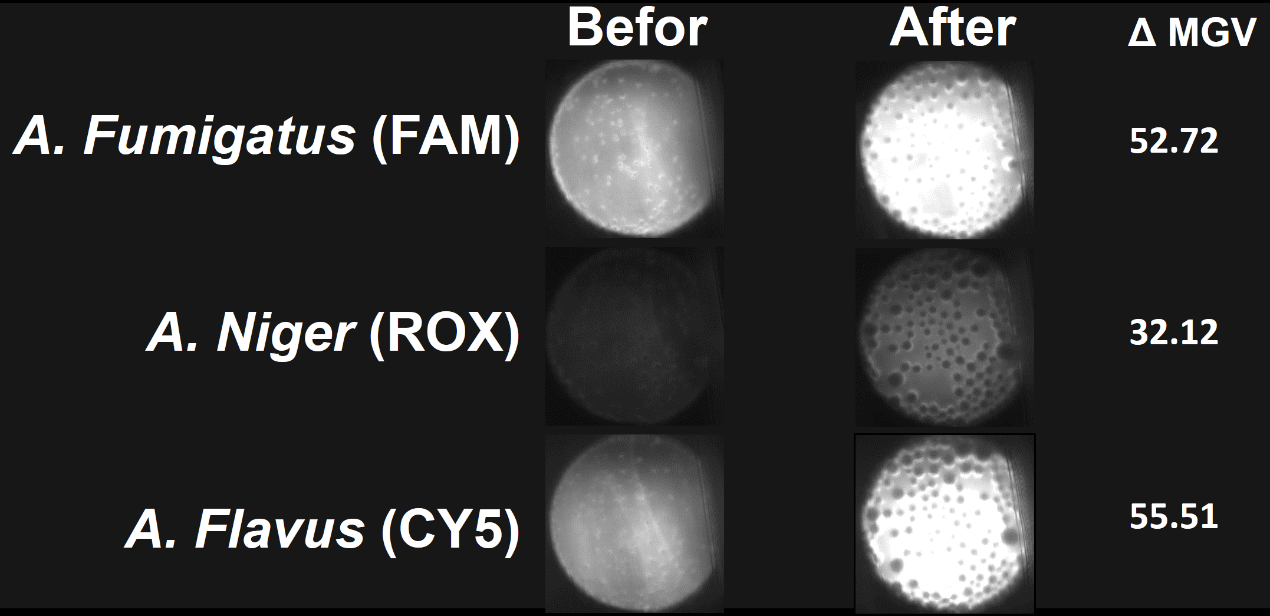
**Fig. S6.** Image of (A) thermal cycle module，(B) fluorescence detection module and (C) chip working on the equipment.

**Fig. S7.** Fluorescence Comparison Images of On-Chip Multiplex TaqMan Assays Before and After Amplification. Δ MGV denotes the difference in the images’ mean gray value before and after PCR.

For the reaction conditions presented in Figure S7, each of the three targets was added at a template concentration of 10⁴ copies per reaction. The concentrations of primers and probes were optimized, and they were added at a ratio of 0.75:1:1.5, corresponding to A. niger, A. fumigatus, and A. flavus respectively. Specifically, a ratio of 1 corresponds to a final concentration of 0.2 μM for probes and 0.4 μM for primers. The concentrations of all other reaction components were consistent with those detailed in Section 2.5 of this manuscript. On-chip PCR amplification was conducted using our self-constructed device, with the following program: initial denaturation at 94 °C for 1 min, followed by 45 cycles of denaturation at 95 °C for 10 s and annealing/extension at 60 °C for 30 s (~30min).


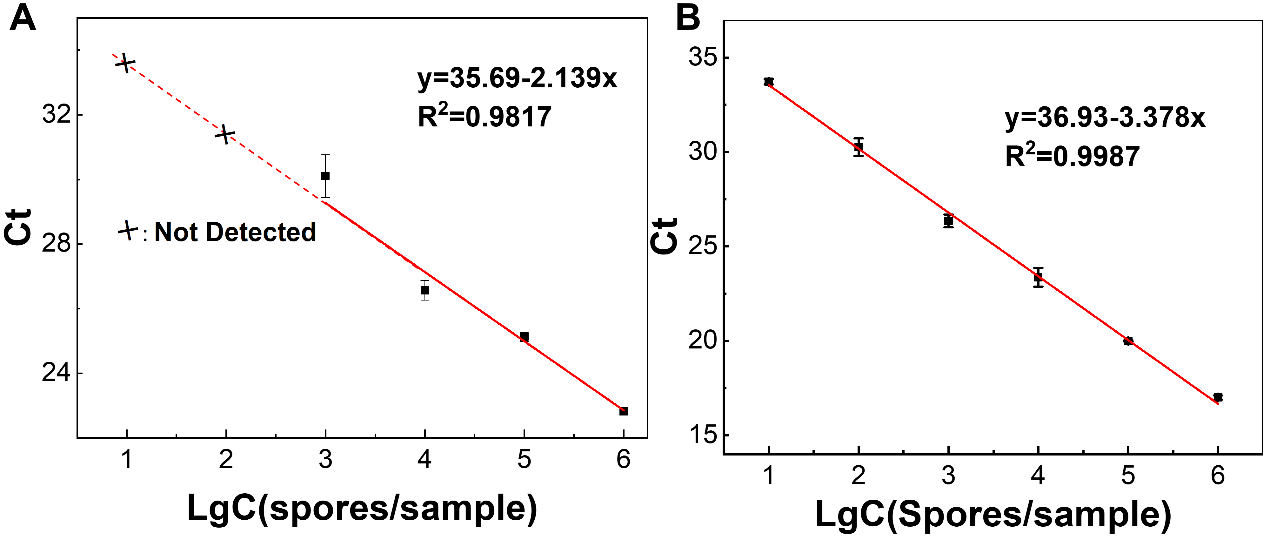


**Fig. S8.** Ct–concentration linearity for *A. niger* at different concentrations using two extraction methods. (A) Qiagen; (B) USBB&MBDA

Samples containing *A. niger* at a concentration of 10⁵ spores per sample were subjected to DNA extraction using a Qiagen commercial kit and USBB&MBDA, respectively. The PCR amplification Ct values obtained from each method were substituted into the corresponding linear equations to calculate the actual number of lysed spores. The ratio of the calculated spore numbers was defined as the lysis efficiency of the ultrasonic method, assuming that the commercial kit achieved 100% lysis efficiency with *Ct2*.

$$Spore lysis rate=\left( 1-{10}^{(\frac{32.93-Ct1}{3.52}-\frac{32.93-Ct2}{3.52})} \right)\times100\%$$

*Ct1*: USBB&MBDA;

*Ct2*: Qiagen commercial kit

Based on the above calculation, the lysis efficiency of the ultrasonic method for *A. niger* at a concentration of 10⁵ spores per sample was determined to be 85.5%, corresponding to *Ct2*.


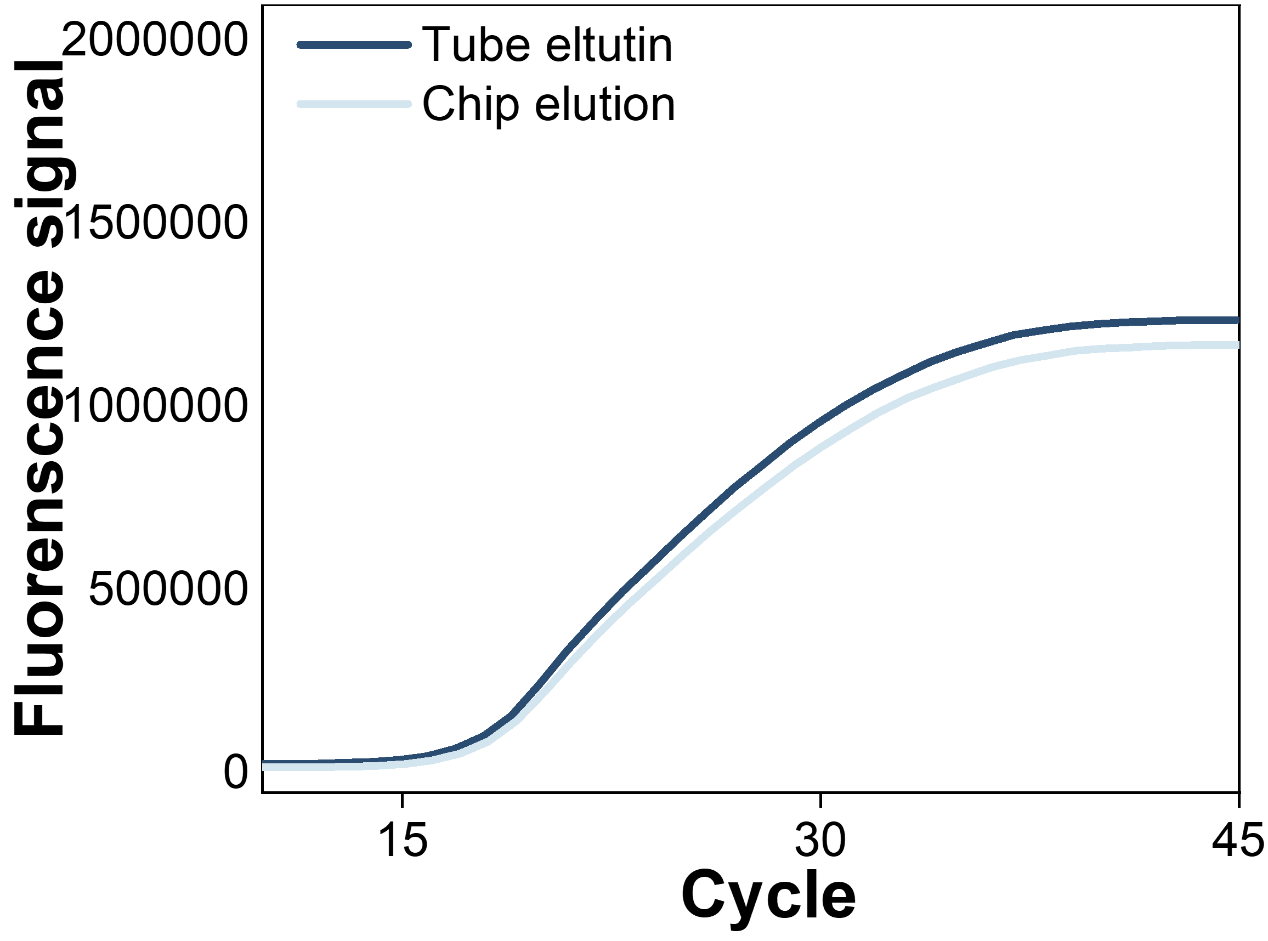


**Fig. S9.** Loss of magnetic beads during traversal across oil-water interface in chip

To investigate the potential loss of magnetic beads induced by repetitive traversal across the oil-water interface in a chip, *A. niger* spores at a concentration of 10⁶ spores/test were used as the target. Spore lysis was performed following the USBB method described in Section 2.5. Subsequent magnetic bead washing procedures were conducted in test tubes and chips, respectively. The amplification chambers of the chips were filled with 100μL TE buffer; after the magnetic beads were transferred into the chambers, the entire mixture of liquid and MBs was transferred to 1.5 mL tubes. Nucleic acids were then eluted into 100 μL TE buffer, and PCR was performed according to the method described in Section 2.3. The Ct values obtained from PCR amplification of each group were substituted into the corresponding linear equations (Fig. 2I) to calculate the respective nucleic acid concentrations. The loss rate was defined as the difference between 1 and the ratio of these two concentration values.

$$Loss rate=\left( 1-{10}^{(\frac{32.93-\bar{Ct4}}{3.52}-\frac{32.93-\bar{Ct3}}{3.52})} \right)\times100\%$$

Tube elution：= 18.258±0.038 (18.263; 18.293; 18.217);

Chip elution：= 18.495±0.022 (18.495; 18.537; 18.529);

Based on the aforementioned calculations, the repetitive traversal of magnetic beads across the oil-water interface in chips resulted in a final loss rate of 14.36% ± 2.46 %.


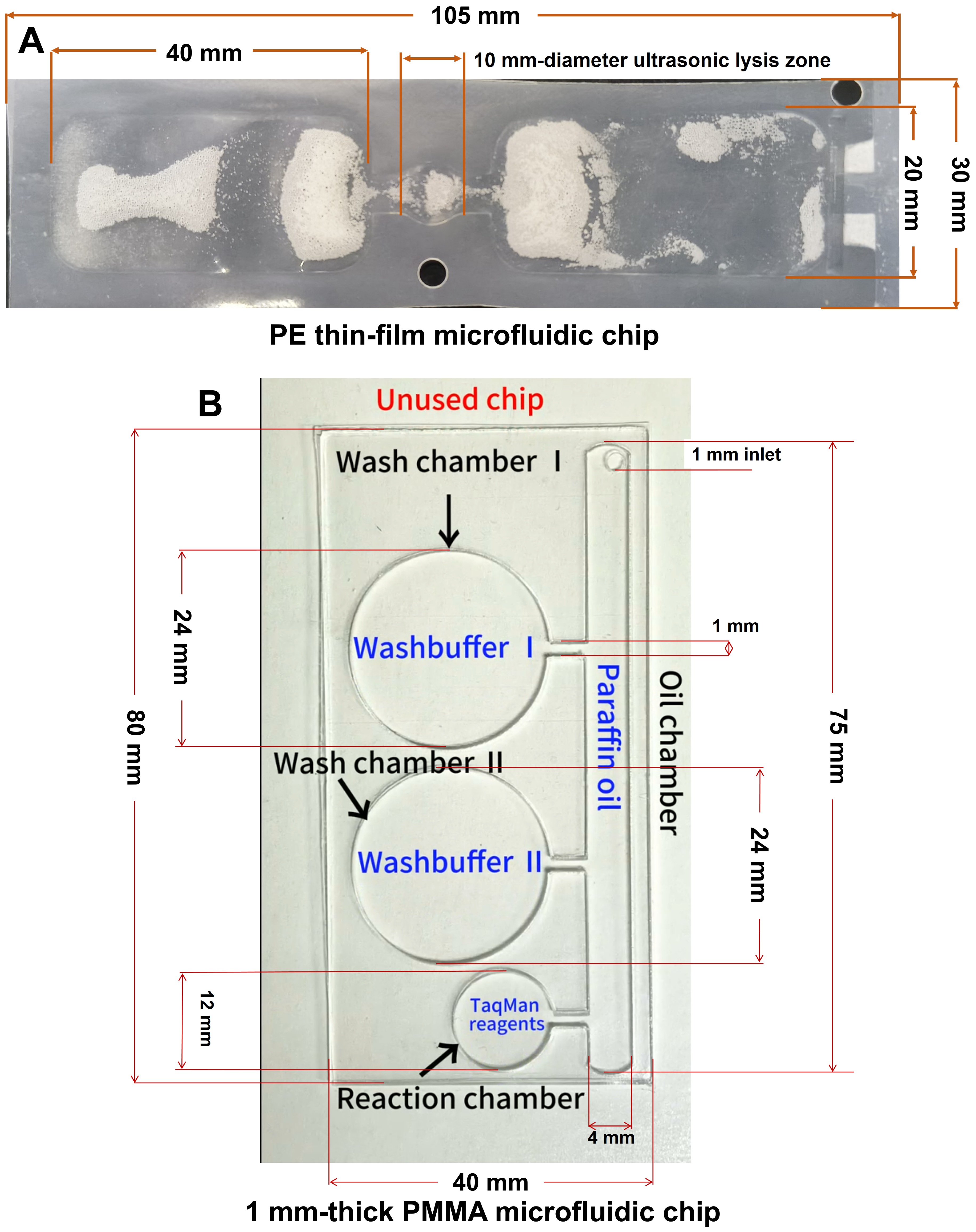


**Fig. S10.** Schematic diagram of the structure and parameters of the thin-film chip (A) and microfluidic chip (B).


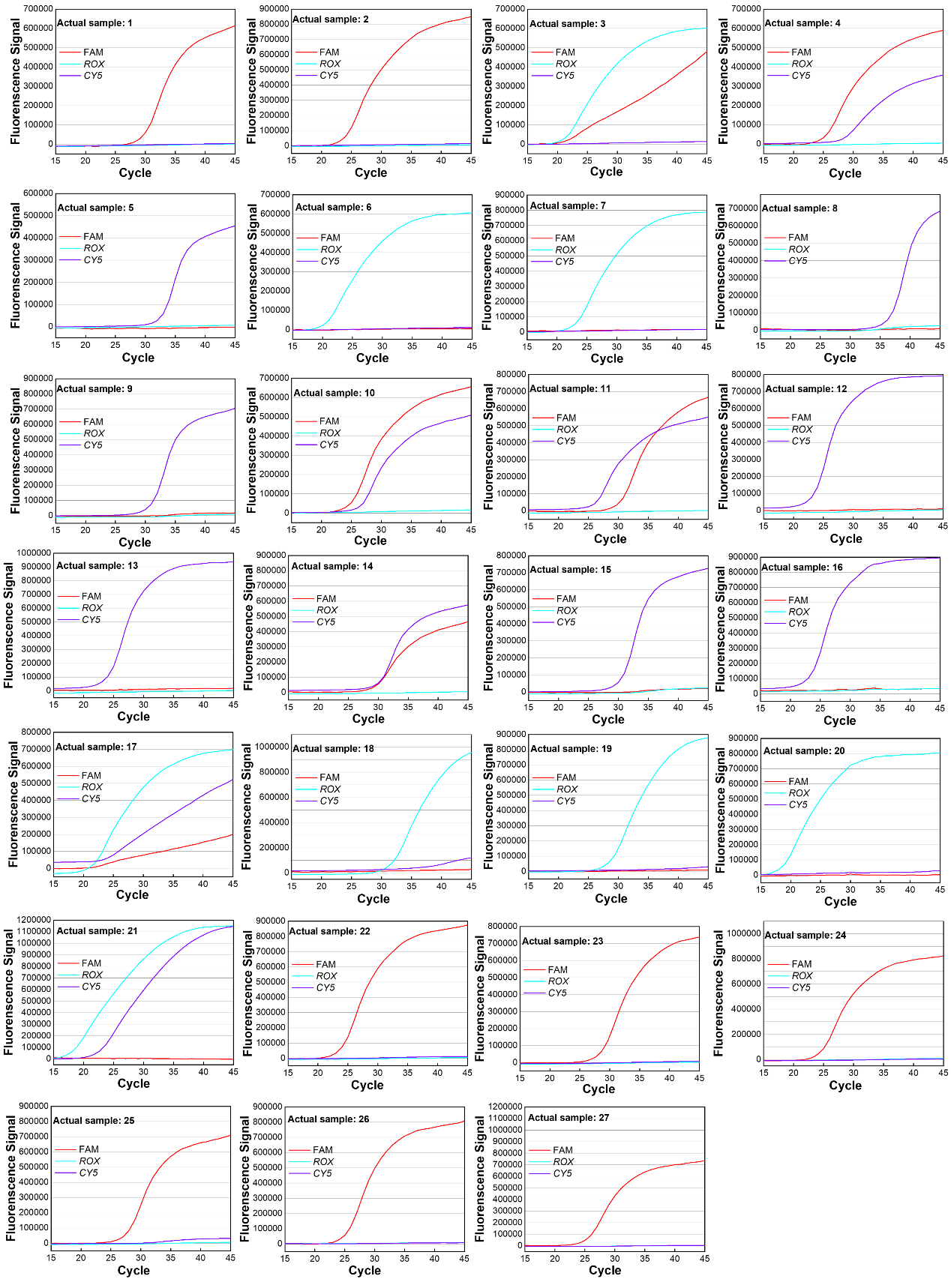


**Fig. S11.** Detection results of 27 positive actual samples
